# Using smartphone battery data to infer sleep-wake metrics in psychiatric cohorts – an exploratory study

**DOI:** 10.1192/j.eurpsy.2022.746

**Published:** 2022-09-01

**Authors:** S. Howes, G. Gillett, N. Palmius, A. Bilderbeck, G. Goodwin, K. Saunders, N. Mcgowan

**Affiliations:** 1 John Radcliffe Hospital, Oxford University Clinical School, Oxford, United Kingdom; 2 King’s College London, Institute Of Psychiatry, Psychology & Neuroscience, London, United Kingdom; 3 University of Oxford, Institute Of Biomedical Engineering, Oxford, United Kingdom; 4 Manor House, P1vital Products, Wallingford, United Kingdom; 5 University of Oxford, Department Of Psychiatry, Oxford, United Kingdom

**Keywords:** digital phenotyping

## Abstract

**Introduction:**

Disturbances to sleep-wake patterns are associated with bipolar disorder (BD) and borderline personality disorder (BPD). Objective assessment typically involves actigraphy monitoring, although it may be possible to derive sleep-wake metrics from other digital data, such as smartphone battery degradation.

**Objectives:**

To assess whether common actigraphy-derived phase markers of the sleep-wake pattern (L5 and M10 onset) are in agreement with measures derived from smartphone battery data and explore if battery metrics differ between people with BD, BPD , and a healthy control group (HC).

**Methods:**

High frequency smartphone battery data was collected from 30 BD, 19 BPD and 33 HC participants enrolled in the Automated Monitoring of Symptom Severity (AMoSS) study, over 28 days. Participants also wore an actigraph during this period. L5 and M10 values were calculated separately based on the rate of smartphone battery degradation and conventional actigraphy methods. Bland-Altman analyses were performed to assess agreement between battery-derived and actigraphy-derived values, and Kruskal-Wallis tests used to compare diagnostic groups.

**Results:**

For L5, battery-derived and actigraphy-derived values had a bias of 0.46 [-0.10, 1.02], upper limit of agreement (LOA): 5.45 [4.49, 6.41], and lower LOA: -4.53 [-3.56, -5.49]. For M10, the bias was 0 [-0.92, 0.92], upper LOA: 8.19 [6.61, 9.76], and lower LOA: -8.19 [-6.61, -9.76]. Between diagnostic groups, there was no difference for battery-derived M10 (p=0.652), or L5 (p=0.122).

**Conclusions:**

Our results suggest battery-derived and actigraphy-derived M10 and L5 show good overall equivalence. However, battery-derived methods exhibit large variability, which limits the clinical utility of smartphone battery data to infer sleep-wake metrics.

**Disclosure:**

No significant relationships.

